# Outcomes of internal and external fixation in midfoot Charcot neuroarthropathy: a systematic review and single-arm meta-analysis

**DOI:** 10.1186/s13018-026-07144-0

**Published:** 2026-07-30

**Authors:** Karthik Chinnaswamy, Ephraim Khimbele, Ahmed AlSaggaf, Karim Awad, Abdul-Hadi Kafagi, Upamanyu Nath, Justin Mooteeram, Anand Pillai

**Affiliations:** 1 Faculty of Biology, Medicine and Health, The University of Manchester, Manchester, M13 9PL UK; 2 Royal Preston Hospital, PR2 9HT Preston, England, UK; 3 St Mary’s Hospital, London, W2 1NY England, UK; 4 Department of Trauma and Orthopaedics, Wythenshawe Hospital, Manchester, M23 9LT England, UK

**Keywords:** Charcot neuroarthropathy, Diabetic foot, Midfoot, External fixation, Internal fixation, Reconstruction, Surgery, Systematic review, Meta-analysis

## Abstract

**Background:**

Midfoot Charcot neuroarthropathy (CN) reconstruction is often performed using internal fixation (IF) or external fixation (EF). This systematic review summarizes clinical outcomes of these techniques including fusion, amputation, return to ambulation and complication profiles.

**Methods:**

A PRISMA-guided systematic review identified studies of midfoot CN reconstruction using IF or EF. Random-effects single-arm meta-analyses of logit-transformed proportions were used to pool postoperative amputation and fusion; fixation type was explored using mixed-effects meta-regression. Robustness was evaluated using leave-one-out analyses, influence diagnostics and patient-limited reweighting for bilateral reconstructions. Small-study effects were assessed with Egger’s regression.

**Results:**

Seventeen observational studies were included. The pooled postoperative amputation rate was 8.7% (95% CI 5.8–12.9; PI 3.6–19.7; k = 15), with meta-regression showing no significant association with fixation type (*p* = 0.163). Subgroup estimates were 10.2% after IF (95% CI 5.6–17.7) and 6.4% after EF (95% CI 5.1–7.9). Fusion pooled at 80.2% (95% CI 59.9–91.7; PI 17.5–98.7; k = 11), with substantial heterogeneity not explained by fixation type (*p* = 0.631). Subgroup pooling yielded fusion estimates of 81.5% (95% CI 59.8–92.9; k = 9) for IF and 75.6% (95% CI 0–100; k = 2) for EF, noting imprecision for EF due to limited studies. Return to ambulation was 94.1% in IF and 81.3% in EF. Infection, hardware failure, and revision rates were 21.8%, 31.6%, and 31.7% in IF, and 5.7%, 12.5%, and 19.1% in EF, respectively. Patient-limited reweighting produced near-identical pooled estimates (amputation 8.9%; fusion 79.9%).

**Conclusion:**

Midfoot CN reconstruction generally achieves high limb salvage and fusion overall. When stratified by fixation category, ambulation and fusion were higher in IF, whereas amputation, infection, hardware failure, and revision rates were also higher. Between-category differences may reflect confounding by indication and should be interpreted as hypothesis-generating. These findings support patient-tailored fixation selection and suggest that differences largely reflect multiple patient factors rather than a causal effect of fixation type. Fixation choice is also likely to be influenced, to some extent, by surgeon expertise and familiarity with specific reconstructive techniques.

**Supplementary Information:**

The online version contains supplementary material available at https://doi.org/10.1186/s13018-026-07144-0.

## Introduction

Charcot neuroarthropathy (CN) is a neuropathic, inflammation-driven disorder that causes rapid destruction and destabilization of foot joints in people with peripheral neuropathy [1]. 

The midfoot is most commonly affected site, accounting for up to 77% of cases and predisposing to plantar bony prominence, ulceration, and recurrent infection [2]. Once deformity and instability are established, midfoot CN is strongly linked to chronic ulceration and, in turn, to excess amputation and mortality risk, making prevention of recurrent breakdown a central goal [2, 3]. 

Charcot midfoot reconstruction has evolved along two principal fixation philosophies. Internal fixation uses rigid constructs such as plates, screws, and intramedullary “beaming” along the medial and lateral columns. External fixation, often with circular or hexapod frames, enables gradual deformity correction, powerful off-loading, and staged care without reliance on definitive internal implants [4]. Each approach carries trade-offs: internal constructs risk hardware failure, non-union; frames can entail pin-site infection, patient intolerance, and prolonged treatment courses. Consensus on when to prefer one strategy remains elusive [5]. 

Despite expanding surgical adoption, the evidence guiding choice of fixation is limited and heterogeneous. Prior systematic reviews, often pooling different anatomic regions and combining various constructs have largely comprised level IV case series with wide variability in case mix, staging, and outcome definitions. Conclusions have therefore been inconsistent and non-definitive. We anticipated that observed patterns would primarily reflect confounding by indication rather than inherent construct superiority.

Accordingly, the aim of this systematic review and single-arm meta-analysis was to evaluate outcomes of internal and external fixation in adult patients undergoing reconstruction for midfoot Charcot neuroarthropathy, including amputation, fusion, return to ambulation, and complication profiles. Because the available evidence was observational, any between-category differences were interpreted as observed patterns rather than direct comparative evidence of superiority.

## Methods

This systematic review was completed in accordance with the Preferred Reporting Items for Systematic Reviews and Meta-Analyses (PRISMA) guidelines. This systematic review was registered at the International Prospective Register of Systematic Reviews (PROSPERO – CRD420251006055). The review protocol can be accessed through PROSPERO.

### Search strategy

A systematic literature search was completed across four databases: PubMed, CINAHL, Scopus, and the Cochrane Library. The search period was restricted to articles published between January 2000 and April 2025. Search terms covered two domains: (1) Charcot-related conditions and (2) reconstructive surgical techniques and fixation methods. Specific search strings are detailed in the Appendix.

Literature was managed using the Rayyan platform (www.rayyan.ai*).* A two-stage screening process was conducted independently by two authors, who evaluated titles and abstracts, followed by a detailed full-text review. Any conflicts regarding study eligibility were resolved through consensus-based discussion; if a resolution could not be reached, a third senior reviewer was consulted.

### Eligibility criteria

Studies were considered eligible for inclusion if they involved adult populations (> 18 years) diagnosed with Charcot Neuroarthropathy (CN) of any etiology, provided the pathology specifically affected the midfoot. Eligible manuscripts were required to detail surgical reconstruction utilizing internal and/or external fixation methods. Inclusion was limited to cohorts consisting of at least 10 patients undergoing surgical reconstruction affecting any part of the foot or ankle for CN. Additionally, studies were required to report a minimum mean postoperative follow-up of 12 months and document at least one postoperative clinical or radiographic outcome.

Studies were excluded if published in languages other than English, as well as case reports, series with fewer than 10 patients, and cadaveric or biomechanical investigations. Furthermore, research focusing solely on conservative management, isolated exostectomies, osteotomies, or soft-tissue procedures without a primary reconstructive component was excluded from this analysis.

### Primary outcomes

This review focused primarily on two key outcomes following surgical reconstruction for midfoot Charcot neuroarthropathy (CN): postoperative amputation and successful osseous fusion. These endpoints were chosen because they reflect the core objectives of CN management: limb preservation, achievement of a stable, plantigrade foot, and minimization of further complications.

### Secondary outcomes

Secondary outcomes in this review encompassed a range of functional and safety-related measures, including restoration of ambulation, which was defined as any postoperative walking, development of ulcerations, postoperative infections (i.e., osteomyelitis, septic arthritis, hardware related infection), pin site infections (confined to skin and subcutaneous tissue), complications related to hardware (such as loosening, migration, or failure), and the necessity for revision procedures. These variables were selected to provide a comprehensive evaluation of patient recovery, complication incidence, and the overall safety of both internal and external fixation approaches.

All outcomes were based on clinical assessment, with each included study reporting a mean follow-up period exceeding 12 months, thereby offering sufficient duration to capture relevant postoperative events and recovery trajectories.

### Data extraction and management

Data collected included study characteristics (author, year, country), fixation technique details, cohort characteristics (sample size, demographics, follow-up), and all prespecified primary and secondary outcomes. Two reviewers independently performed extraction, with discrepancies resolved by discussion and, when required, by a third senior reviewer.

### Data synthesis

A meta-analysis of proportions was performed to estimate the pooled outcome rates following Charcot reconstruction. Proportions were logit-transformed prior to pooling to stabilize variance and account for studies reporting proportions near 0 or 1. A random-effects model was applied using restricted maximum likelihood (REML) estimation.

Hartung-Knapp adjustment was applied to provide more robust confidence intervals given the relatively small number of included studies. Between-study heterogeneity was quantified using Cochran’s Q statistic, τ², and the I² statistic.

To evaluate expected dispersion of treatment effects in future clinical settings, a 95% prediction interval was calculated.

Subgroup differences between internal and external fixation techniques were examined using mixed-effects meta-regression with fixation type entered as a categorical moderator.

All analyses were performed in R (version 4.5) using the *metafor* package.

Only amputation and fusion were formally pooled using random-effects meta-analysis. Infection, ulceration, hardware failure, revision surgery, pin-site infection and return to ambulation were summarized as descriptive aggregate proportions because definitions and reporting were too heterogeneous for formal meta-analytic synthesis.

Unless otherwise stated, outcomes were analyzed on a limb-based basis, as most included studies reported events at the level of the reconstructed foot rather than the patient. A patient-limited reweighting sensitivity analysis was undertaken to assess the potential influence of bilateral reconstructions.

### Quality assessment

Risk of bias was assessed using the Risk of Bias in Non-Randomized Studies of Interventions (ROBINS-I) tool. Two reviewers independently evaluated the risk with conflicts resolved via a third reviewer.

A formal certainty-of-evidence assessment was not undertaken because the included evidence consisted almost entirely of heterogeneous observational case series with substantial variation in patient selection, fixation constructs, outcome definitions, and follow-up.

### Small-study effects

Small-study effects were assessed using funnel plots and Egger’s regression test. A p-value < 0.05 was interpreted cautiously as possible evidence of small-study effects.

### Sensitivity analyses

Robustness of pooled estimates was evaluated using leave-one-out analyses, with changes in the pooled estimate and heterogeneity (τ², I²) recorded. Study influence and outliers were assessed using studentized residuals, Cook’s distance, DFFITS, leverage (hat values), deletion diagnostics, and Baujat plots. To minimize potential overweighting from bilateral reconstructions, an additional sensitivity analysis was undertaken where patient counts were available by inflating within-study variances by the limbs-to-patients ratio (N_limbs/N_patients) and comparing results with the primary limb-based analyses.

## Results

### Studies

**Fig. 1:**
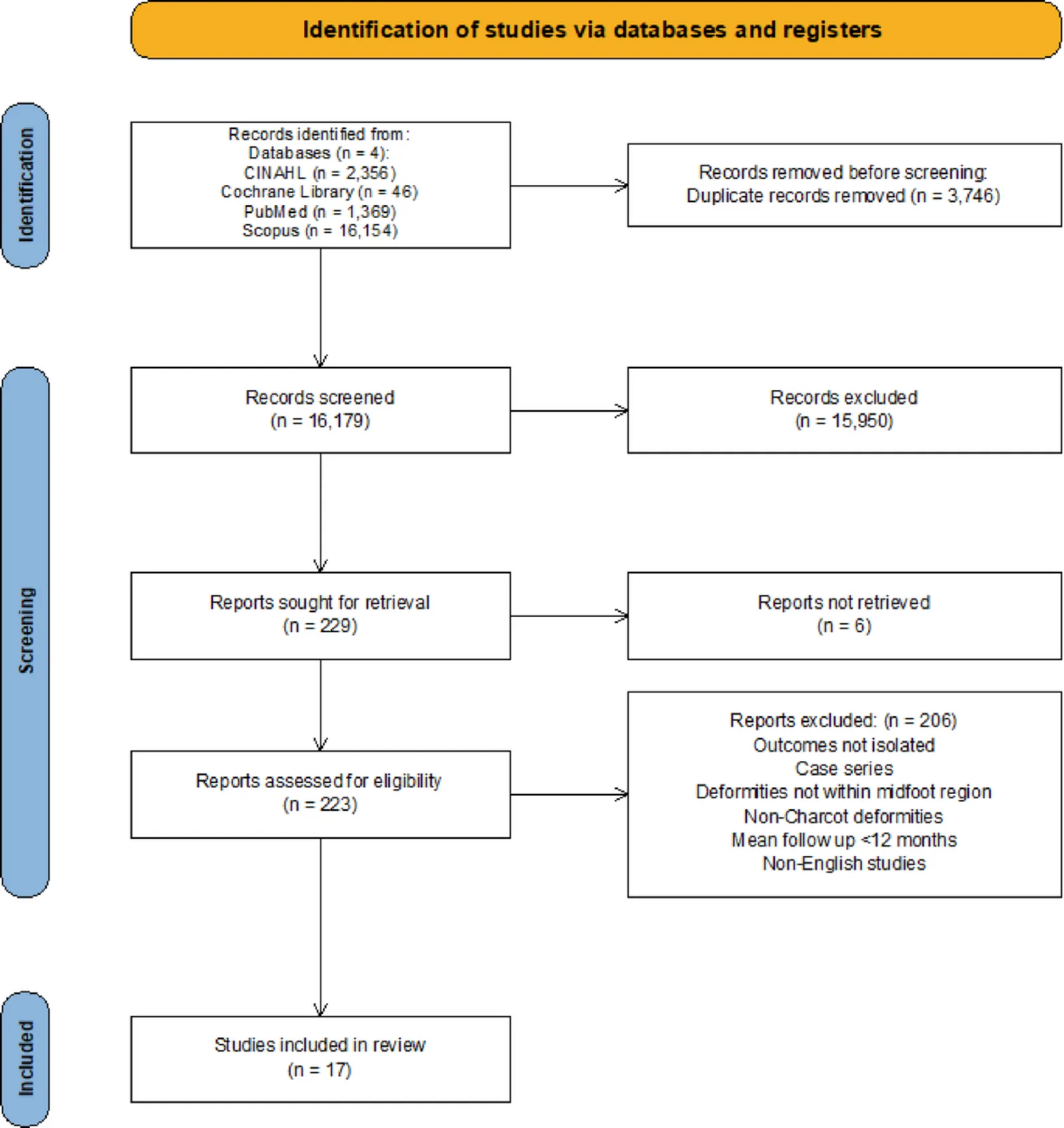
PRISMA diagram

A total of 19,925 reports were recovered using our systematic search terms. Following the initial abstract review, 229 articles were identified for the full review, of which 17 fit our selection criteria. Eleven articles reported on IF and 6 reported on EF (Fig. 1). A supplementary table has been provided to show which studies contributed to each outcome analysis. Studies not included for a given outcome either did not report that outcome or reported it in a form that did not match the prespecified outcome definition.

### Baseline characteristics

A total of 17 studies were included, encompassing 642 patients (656 feet): 350 in the internal fixation (IF) group, and 292 in the external fixation (EF) group.

Baseline patient characteristics are summarized in Table 1 below.

**Table 1:** Baseline characteristics

Author, year, country	Patients (feet)	Mean patient age (years)	Gender (M/F)	Mean follow-up (months)	Fixation Type
Assal et al., 2009, Switzerland [6]	15 (15)	54.9 ± 10.5	9/6	41.5 ± 12.3	Medial column screw
Eschler et al., 2015, Germany [7]	19 (21)	58.8 ± 8.5	14/5	48	Internal corrective arthrodesis
Farber et al., 2002, USA [8]	11(11)	55.0 ± 8.3	NR	24	Skeletal fixator
Ford et al., 2019, USA [9]	25 (25)	58	13/12	18	Beaming
Garchar et al., 2013, USA [10]	24 (25)	58.8 (42–74)	14/10	38.0 (17–64)	Plantar plate
Kroin et al., 2018, USA [11]	25 (25)	59.5	16/9	> 12 mo	Circular
Loveland et al., 2024, USA [12]	30 (30)	59.7	23/7	42	IMN
Manchanda et al., 2020, USA [13]	30 (30)	54.5 ± 8.5	16/14	15.5 ± 6.0	IM screws
Matsumoto et al., 2015, USA [14]	10 (11)	52.2 ± 9.8	4/7	28.9 ± 13.4	MAC fixator
Mehlhorn et al., 2016, Germany [15]	14 (14)	59 ± 9	9/5	21.4 ± 14.6	Fusion bolt & lag screws
Mehlhorn et al., 2019, Germany [16]	40 (40)	61 ± 9	27/13	12 mo	Fusion bolts
Pinzur et al., 2007, USA [17]	26 (26)	56.6	14/12	> 12 mo	Circular
Pinzur et al., 2018, USA [18]	214 (223)	NR	NR	> 12 mo	Circular
Regauer et al., 2023, Germany [19]	84 (84)	59 ± 9	63/21	> 12 mo	Screws or plates
Richter et al., 2015, Germany [20]	47 (48)	60.1	28/19	12 mo	Fusion bolts
Rios Ruh et al., 2019, Spain [21]	6 (6)	56.0 ± 13.7	5/1	17.3 ± 4.6	Circular
Sammarco et al., 2009, USA [22]	22 (22)	59.4 ± 10.9	6/16	52.9 ± 25.5	IMN

### Methods of surgical fixation

Reconstruction was performed using external fixation alone in 302 feet and internal fixation in 354 feet. These should be understood as broad fixation categories rather than uniform interventions. Fixation techniques varied across studies. Internal fixation included plates/screws and intramedullary constructs; external fixation included circular/hexapod and monolateral frames. No association was observed between fixation method and year of publication.

### Complication profiles

#### Amputation rates

**Fig. 2:**
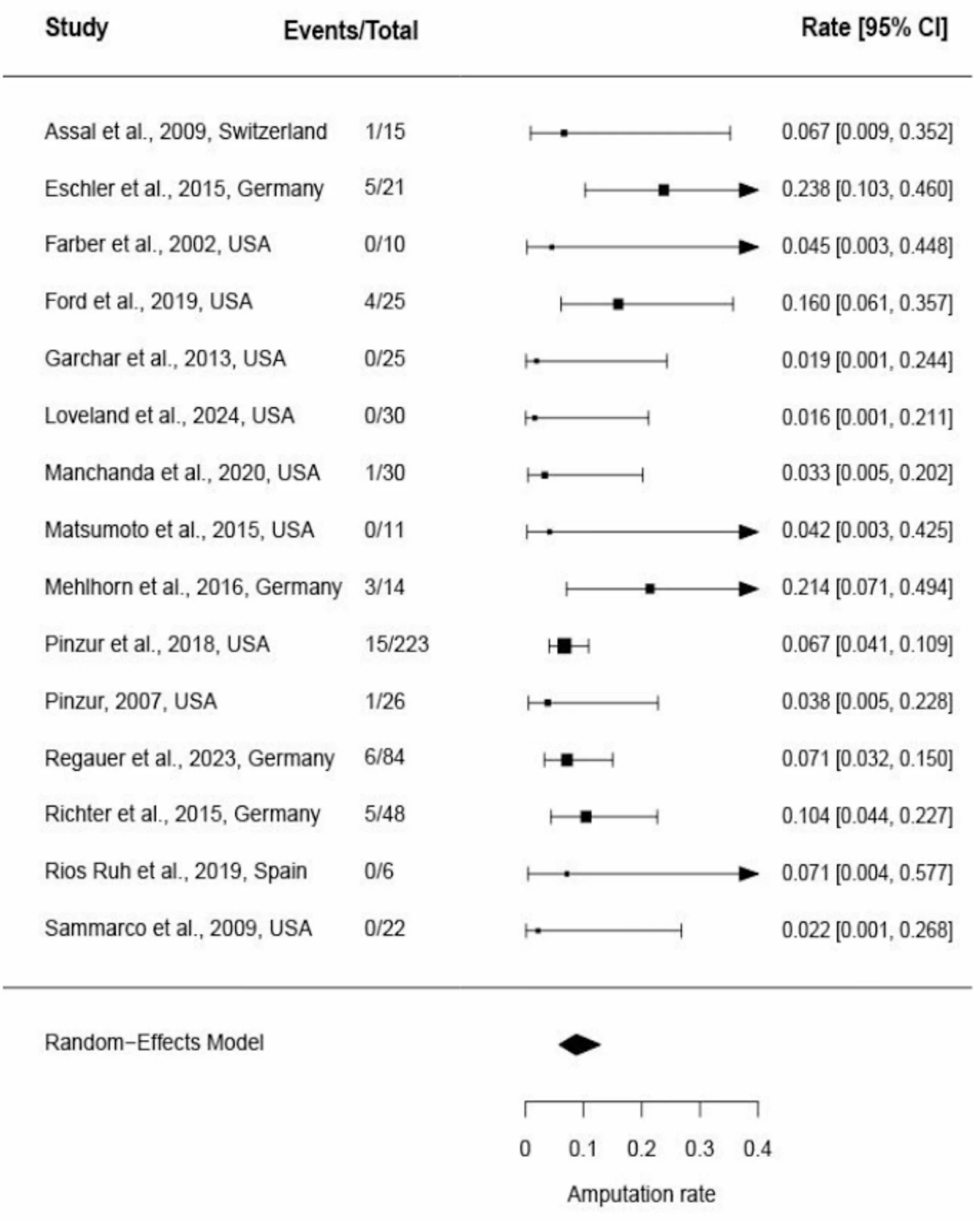
Forest plot of amputation rates

A random-effects meta-analysis of 15 studies produced a pooled logit event rate of − 2.35 (SE 0.20), corresponding to a back-transformed pooled amputation rate of 0.087 (8.7%). The 95% confidence interval (CI) was 0.058 to 0.129, and the prediction interval (PI) ranged from 0.036 to 0.197, indicating that although the average amputation rate is low, rates may vary across clinical settings. Between-study heterogeneity was low to moderate (τ² = 0.152; I² = 25.9%), and the heterogeneity test was not statistically significant (Q(14) = 17.24, *p* = 0.243). Meta-regression comparing internal vs. external fixation did not identify a significant moderator effect (F(1,13) = 2.19, *p* = 0.163), although fixation type accounted for 39.9% of between-study heterogeneity (R² = 39.9%) and residual heterogeneity remained low (I² = 14.2%). Subgroup estimates suggested a pooled amputation rate of 0.102 (10.2%) for internal fixation (95% CI 0.056–0.177) and 0.064 (6.4%) for external fixation (95% CI 0.051–0.079). A patient-limited sensitivity analysis to account for bilateral reconstructions produced an identical pooled estimate (0.089, 95% CI 0.056–0.138) with similar prediction intervals (0.032–0.223 vs. 0.031–0.231). Egger’s regression suggested small-study effects for amputation (*p* = 0.0235). Overall, postoperative amputation appears to be an uncommon outcome in this literature, and variation in amputation risk is unlikely to be explained by fixation choice alone (Fig. 2).

#### Fusion rates

**Fig. 3:**
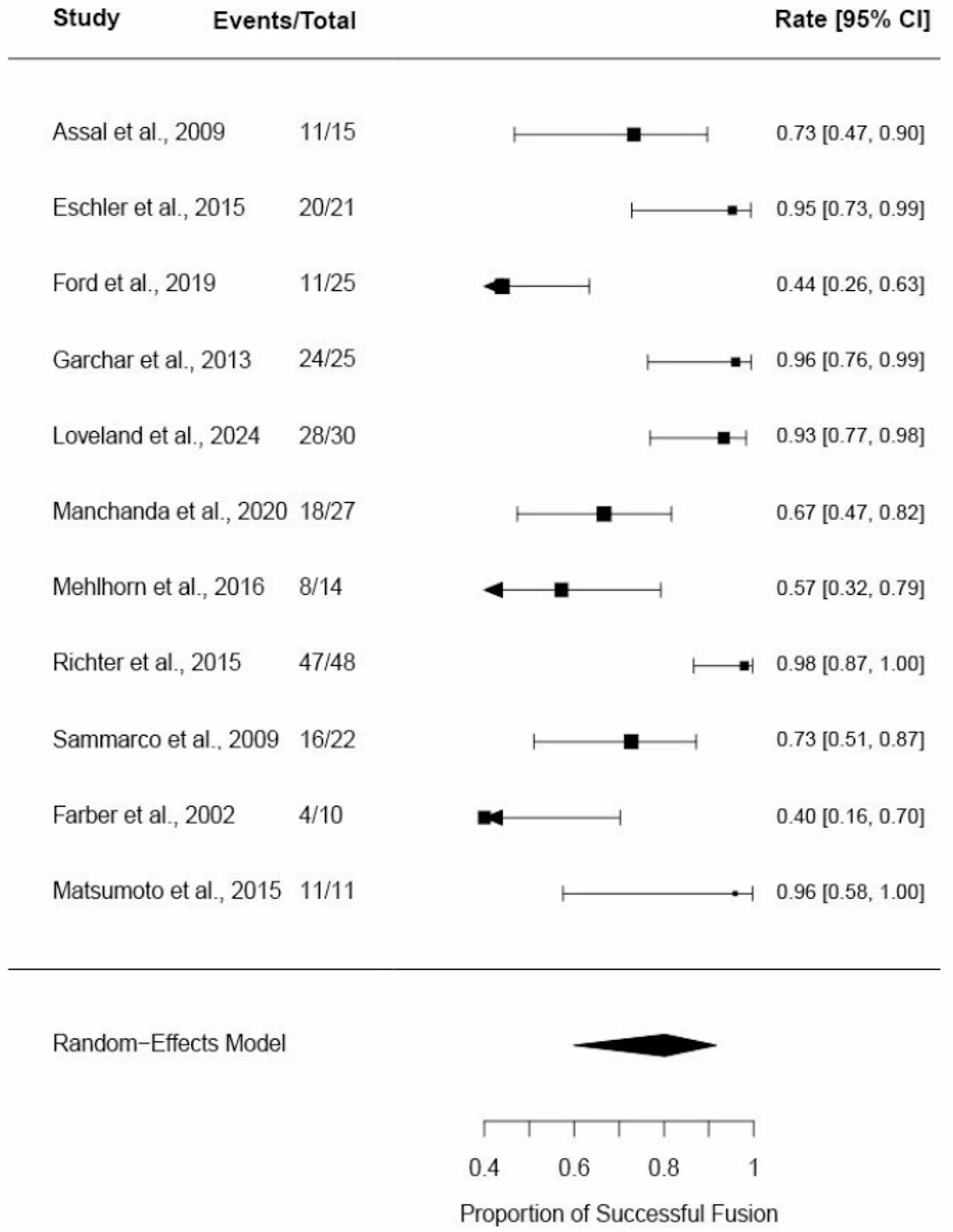
Forest plot of fusion rates

A random-effects meta-analysis of 11 studies produced a pooled logit estimate of 1.40 (SE 0.45), corresponding to a pooled proportion of 0.802 (80.2%). The 95% confidence interval (CI) was 0.599 to 0.917, and the prediction interval (PI) was wide (0.175 to 0.987), indicating substantial variability in expected results across future settings. Between-study heterogeneity was high (τ² = 1.55; I² = 80.4%), and the heterogeneity test was significant (Q(10) = 39.38, *p* < 0.0001). Meta-regression comparing internal vs. external fixation did not identify a significant moderator effect (*p* = 0.6306) and explained 0% of heterogeneity (R² = 0%), with high residual heterogeneity persisting (I² = 81.8%). Subgroup estimates suggested a pooled fusion rate of 0.815 (81.5%; 95% CI 0.598–0.929; k = 9) for internal fixation and 0.756 (75.6%; 95% CI 0.000–1.000; k = 2) for external fixation, noting substantial imprecision for external fixation due to limited study numbers. Patient-limited reweighting to account for bilateral reconstructions produced a near-identical pooled fusion estimate (0.799; 95% CI 0.595–0.915), and subgroup estimates were unchanged (internal 0.813; external 0.749). Fusion was assessed at variable postoperative timepoints across the included studies, and these timings are summarized in the Supplementary Material. This variability in assessment timing likely contributed to the observed heterogeneity in fusion estimates. These findings indicate that fusion outcomes vary considerably across studies and are not explained by fixation approach alone (Fig. 3).

#### Return to ambulation

Return to ambulation was analyzed across 13 studies, including 452 feet. IF showed the highest and most consistent aggregate rate, with an overall return to ambulation of 94.1%, compared with 81.3% for EF (Table 2).

**Table 2:** Return to ambulation

	External fixation	Internal fixation
No. of Studies	6	7
Return to Ambulation, % (n/N) (Feet(	81.3% (244/300)	94.1% (143/152)

#### Soft tissue complications

IF demonstrated higher aggregate rates of infection (21.8%) and ulceration (12.5%), while EF showed lower rates of infection (5.7%) and ulceration (9.1%), while pin-tract infection was reported in only two EF studies as a complication specific to frame-based treatment. Results are summarized in Table 3.

**Table 3:** Soft tissue complications

		External fixation	Internal fixation
Ulceration	No. of studies	5	8
	Percentage (%) (Feet)	9.1% (25/276)	12.5% (25/200)
Infection	No. of studies	4	9
	Percentage (%) (Feet)	5.7% (3/53)	21.8% (63/289)
Pin-tract infection	No. of studies	2	NR
	Percentage (%) (Feet)	17.6% (3/17)	NR

#### Hardware failure and revision

IF demonstrated higher aggregate rates of hardware failure (31.6%) and revision surgery (31.7%), while EF showed lower rates of hardware failure (12.5%) and revision (19.1%). Results are summarized in Table 4.

**Table 4:** Hardware failure and revision rates

		External fixation	Internal fixation
Hardware failure	No. of studies	2	10
	Percentage (%) (Feet)	12.5% (2/16)	31.6% (104/329)
Revision surgeries	No. of studies	3	9
	Percentage (%) (Feet)	19.1% (9/47)	31.7% (99/312)

### Sensitivity analyses

For fusion, the same influence diagnostics identified Richter et al. [20] as the most influential contributor to heterogeneity, with Ford et al. [9] and Farber et al. [8] showing moderate influence. Leave-one-out analyses demonstrated that sequential omission of individual studies did not materially alter the pooled estimate or its statistical significance, and heterogeneity remained substantial across iterations, indicating that variability was not driven by a single outlying study. A patient-limited reweighting approach to account for bilateral reconstructions produced near-identical pooled and subgroup estimates, supporting the robustness of the primary findings.

## Discussion

The decision to use internal versus external fixation in Charcot neuroarthropathy reconstruction is influenced by multiple clinical and practical variables, including deformity complexity, infection status, bone integrity, and overall patient health. Surgeon expertise also plays a significant role. Given the high-risk and unpredictable nature of Charcot pathology, fixation strategies must be individualized to optimize outcomes and minimize complications.

Because all included studies were observational and fixation strategy was determined clinically rather than randomly assigned, comparisons between internal and external fixation were interpreted as observed between-category differences only and not as evidence of treatment superiority.

### Fusion rates

Reported fusion rates were generally favorable with contemporary reconstructive techniques. The pooled fusion rate in this review was 80.2%, with no statistically significant difference between fixation methods, suggesting that factors beyond fixation type influence union.

Metabolic and systemic factors influenced outcomes. Higher HbA1c levels correlated with increased postoperative infection and impaired bone healing, while smoking correlated with non-union and ulcer recurrence. Advanced deformity was associated with lower fusion rates and greater mechanical failure [7, 19]. 

Soft-tissue status was consistently important. Preoperative ulceration and osteomyelitis negatively affected consolidation, and poor bone quality contributed to instability [9, 15]. 

Radiographic union varied across studies. Several reports emphasized that complete radiographic fusion was not always necessary for functional success. Many patients achieved a stable, plantigrade foot sufficient for ulcer healing and long-term limb preservation despite incomplete osseous consolidation [8, 9, 21]. Overall, successful outcomes depended on mechanical stability, biological optimization, and metabolic control rather than fixation method alone.

### Complications and amputation

Complications following CN reconstruction included ulceration, infection, hardware failure, and revision surgery. Predictors of adverse outcomes included preoperative ulceration, poor glycemic control, advanced deformity, and construct insufficiency. Multiple studies in this review correlated higher comorbidity burden with increased infection risk and impaired healing [7, 9, 15, 19, 20]. 

Amputation rates varied across studies, influenced primarily by infection, ulceration, deformity severity, and construct durability. Internal fixation in high-risk or late-stage CN carried the highest amputation risk: 21% in fusion bolt series [15], 24% in late corrective arthrodesis [7], and 13% in intramedullary beaming cohorts [9]. In contrast, contemporary intramedullary devices reported no amputations [12], as did several external fixation series [8, 21]. Only one major amputation occurred among 26 high-risk patients in the neutral ring fixation cohort [17]. 

Amputation rates are notably higher among patients who develop post-operative ulceration or infection. Eschler et al. [7] reported that their cohorts results were in line with a previous study by Saltzman et al. [23] who reported patients without ulceration had an annual amputation risk of 3%, whereas those with ulceration faced a markedly increased risk of 23%. Infection has also been identified as a major contributing factor to amputation. Richter et al. [20] reported all participants who required amputation (*n* = 5) in their study did so as a result of developing infection or sepsis. 

Internal fixation more often reported mechanical and infectious complications. Deep infection occurred in up to 25% of intramedullary beaming cases [9], while early fusion bolt systems showed mechanical failure including bolt loosening or dislocation, as many as 57% of patients [15]. Non-union, hardware breakage, and recurrent ulceration were frequent in osteopenic bone or in the presence of active ulceration. More rigid contemporary devices, such as high-profile bolts and locking intramedullary nails, significantly reduced hardware failure and infection rates [12, 16]. 

External fixation demonstrated a different and often more favorable complication profile. Pin-site infections were common but typically superficial and responsive to simple care [17, 21]. Recurrent ulceration was rare and easily managed, with neutral ring fixation reporting only four recurrences, all resolved with exostectomy [17]. Static circular fixation and staged correction techniques showed low complication burdens even in ulcerated or infected feet [8, 21]. 

The concept of the “superconstruct” has become central in CN reconstruction, emphasizing maximized stability through strategic hardware placement. Manchanda et al. described the superconstruct as extending fixation beyond the zone of injury which resulted in fewer complications and also suggested that increasing the number of fixation points with screws reduces the rate of hardware failure and improves construct durability [13]. 

Internal constructs were more often susceptible to deep infection, hardware failure, and amputation when used in the setting of active ulceration, osteomyelitis, or compromised soft tissue. Earlier beaming and bolt systems exhibited particularly high mechanical complication rates [9, 15]. 

External fixation may offer practical advantages in high-risk or contaminated environments. Circular and multi-axial fixators achieved consistently high limb-salvage rates, including 100% in several cohorts [8, 21], with low deep-infection and ulcer-recurrence rates even in patients with ongoing ulceration or osteomyelitis. While radiographic fusion was less common, functional union and durable correction were reliably achieved with minimal need for major amputation [17]. 

Fixation choice should therefore be guided by ulcer status, infection burden, bone quality, deformity severity, and metabolic control rather than a single preferred technique.

### Limitations

This systematic review is constrained by the quality and heterogeneity of the available evidence. All included studies were retrospective case series, with no randomized or prospective comparative designs, limiting causal inference and increasing susceptibility to selection bias, confounding, and incomplete outcome reporting. Because ROBINS-I identified predominantly moderate-to-serious risk of bias, especially from confounding, any apparent differences between fixation methods must be interpreted cautiously and should not be considered evidence of causal superiority. Accordingly, the overall certainty of evidence for the main outcomes, and particularly for any comparison between fixation methods, should be considered low to very low.

Patient populations were heterogeneous with respect to deformity severity, ulceration and infection status, prior interventions, and comorbidities. Similarly, fixation techniques varied considerably across studies, encompassing differences in construct design, fixation density, adjunctive procedures, and postoperative protocols. This clinical diversity limits the validity of direct comparisons between internal and external fixation strategies and introduces potential confounding by indication, as more complex or infected cases were preferentially managed with external fixation.

Definitions of fusion were inconsistent, with studies variably applying radiographic, CT-based, or clinical criteria. Because stable fibrous union may provide satisfactory functional outcomes in Charcot reconstruction, reliance on radiographic consolidation alone may not accurately reflect clinically meaningful success. Reporting of complications was similarly heterogeneous; few studies differentiated superficial from deep infections or minor hardware revisions from major reoperations, precluding uniform grading of complication severity.

Definitions of return to ambulation were also inconsistent, ranging from independent walking and protected weight-bearing to walking in therapeutic footwear, with no study benchmarking against preoperative mobility. For this review, any form of walking was counted as return to ambulation; because these endpoints capture different functional thresholds, the observed difference between internal and external fixation should be interpreted as definition-dependent reporting rather than a true functional difference.

Key metabolic and systemic risk factors, including glycemic control, nutritional status, and body mass index, were inconsistently reported, limiting the ability to conduct stratified or risk-adjusted analyses. Functional outcomes, such as pain, ambulation, ulcer recurrence, and validated patient-reported measures, were variably reported, further restricting comprehensive outcome assessment. Follow-up duration was often limited to short- and mid-term intervals, potentially underestimating late mechanical failure, recurrent deformity, or ulceration.

Although visual inspection of funnel plots did not show marked asymmetry, formal assessment suggested evidence of small-study effects for the midfoot amputation outcome (Egger-type regression test *p* = 0.0235). This should be interpreted cautiously given logit-transformed proportions and clinical heterogeneity. In addition, the small number of studies within some fixation categories and the limited reporting of key clinical covariates reduce the precision of pooled estimates and constrain the reliability of between-technique comparisons. Consequently, pooled amputation and complication rates may be underestimated, and subgroup analyses should be considered exploratory.

Taken together, these findings must be interpreted within the context of heterogeneous, predominantly level IV evidence. High-quality, prospective, multicenter studies incorporating standardized definitions of fusion and functional success, rigorous reporting of metabolic and systemic risk factors, validated patient-reported outcome measures, and robust adjustment for disease severity are necessary to establish definitive, evidence-based guidance for midfoot Charcot reconstruction.

## Conclusion

This systematic review included 17 observational studies encompassing 642 patients (656 feet). Pooled estimates indicate that limb salvage and osseous fusion were achieved in the majority of cases following midfoot reconstruction for Charcot neuroarthropathy using both internal and external fixation. External fixation was more commonly applied in cases with active infection, compromised soft tissues, or complex deformity, and tended to show lower fusion rates with fewer devastating complications. In contrast, internal fixation showed higher observed fusion rates but also reported higher rates of deep infection, hardware failure, and the need for revision surgery.

Given the clinical and methodological heterogeneity across studies and the likelihood of confounding by indication identified by ROBINS-I, these findings should be interpreted as trends rather than direct comparative evidence rather than evidence of comparative superiority between fixation methods. Internal fixation was more often used in biologically fitter patients with good bone quality and intact soft tissues but risks potentially severe complications. External fixation may be better suited for patients with infection, ulceration, or significant systemic comorbidities, providing safer limb salvage despite sometimes lower observed fusion rates. Ultimately, the key to successful Charcot midfoot reconstruction is selecting the fixation strategy that best aligns with the patient’s biological and clinical profile, rather than applying a single method universally.

## Supplementary Information

Below is the link to the electronic supplementary material.

Supplementary Material 1

Supplementary Material 2

Supplementary Material 3

Supplementary Material 4

Supplementary Material 5

Supplementary Material 6

Supplementary Material 7

Supplementary Material 8

Supplementary Material 9

## Data Availability

Data supporting the findings of this study are available from the corresponding author upon reasonable request.
