# Supplementary material for: Outcomes of internal and external fixation in midfoot Charcot neuroarthropathy: a systematic review and single-arm meta-analysis
Source: J Orthop Surg Res. 2026 Jul 30;21:612. doi: 10.1186/s13018-026-07144-0 (PMC13711758; doi:10.1186/s13018-026-07144-0)
Supplement: Supplementary file 1 [file 13018_2026_7144_MOESM6_ESM.docx]

| **Study** | **Fixation Type** | **Successful Fusion/Union** | **Infection** | **Ulceration** | **Amputation** | **Return to Ambulation** | **Hardware failure** | **Revision surgery** | **Pin Tract Infection** |
| --- | --- | --- | --- | --- | --- | --- | --- | --- | --- |
| Assal et al., 2009, Switzerland | Internal | Reported | Reported | Reported | Reported | Reported | Reported | Reported | NR |
| Eschler et al., 2015, Germany | Internal | Reported | Reported | Reported | Reported | Reported | Reported | Reported | NR |
| Farber et al., 2002, USA | External | Reported | Reported | Reported | Reported | Reported | Reported | Reported | NR |
| Ford et al., 2019, USA | Internal | Reported | Reported | Reported | Reported | Reported | Reported | Reported | NR |
| Garchar et al., 2013, USA | Internal | Reported | Reported | Reported | Reported | Reported | NR | NR | NR |
| Kroin et al., 2018, USA | External | NR | NR | NR | NR | Reported | NR | NR | NR |
| Loveland et al., 2024, USA | Internal | Reported | Reported | Reported | Reported | Reported | Reported | Reported | NR |
| Manchanda et al., 2020, USA | Internal | Reported | Reported | NR | Reported | NR | Reported | Reported | NR |
| Matsumoto et al., 2015, USA | External | Reported | Reported | Reported | Reported | Reported | NR | Reported | Reported |
| Mehlhorn et al., 2016, Germany | Internal | Reported | Reported | Reported | Reported | Reported | Reported | NR | NR |
| Mehlhorn et al., 2019, Germany | Internal | NR | NR | NR | NR | NR | Reported | Reported | NR |
| Pinzur et al., 2018, USA | External | NR | NR | Reported | Reported | Reported | NR | NR | NR |
| Pinzur, 2007, USA | External | NR | Reported | Reported | Reported | Reported | NR | Reported | NR |
| Regauer et al., 2023, Germany | Internal | NR | Reported | NR | Reported | NR | Reported | Reported | NR |
| Richter et al., 2015, Germany | Internal | Reported | Reported | Reported | Reported | NR | Reported | Reported | NR |
| Rios Ruh et al., 2019, Spain | External | NR | Reported | Reported | Reported | Reported | Reported | NR | Reported |
| Sammarco et al., 2009, USA | Internal | Reported | NR | Reported | Reported | Reported | Reported | Reported | NR |
