# Supplementary material for: Outcomes of internal and external fixation in midfoot Charcot neuroarthropathy: a systematic review and single-arm meta-analysis
Source: J Orthop Surg Res. 2026 Jul 30;21:612. doi: 10.1186/s13018-026-07144-0 (PMC13711758; doi:10.1186/s13018-026-07144-0)
Supplement: Supplementary file 2 [file 13018_2026_7144_MOESM7_ESM.docx]

Definitions of return to ambulation per study

| **Study** | **Fixation group** | **RTA Definitions** |
| --- | --- | --- |
| **Mehlhorn et al. 2016** | IF | Return to therapeutic footwear |
| **Sammarco et al. 2009** | IF | Independent walking |
| **Assal et al. 2009** | IF | Return to therapeutic footwear |
| **Ford et al. 2018** | IF | NR |
| **Garchar et al. 2013** | IF | Protected weight bearing |
| **Loveland et al. 2024** | IF | Return to therapeutic footwear |
| **Pinzur et al. 2007** | EF | Return to therapeutic footwear |
| **Matsumoto et al. 2015** | EF | Return to therapeutic footwear |
| **Rios Ruh et al. 2019** | EF | Return to therapeutic footwear |
| **Farber et al. 2002** | EF | Return to therapeutic footwear |
| **Pinzur et al. 2018** | EF | Return to therapeutic footwear |
| **Kroin et al. 2018** | EF | Return to therapeutic footwear |
| **Eschler et al. 2015** | IF | Mixed – Independent walking or therapeutic footwear |
