# Supplementary material for: Outcomes of internal and external fixation in midfoot Charcot neuroarthropathy: a systematic review and single-arm meta-analysis
Source: J Orthop Surg Res. 2026 Jul 30;21:612. doi: 10.1186/s13018-026-07144-0 (PMC13711758; doi:10.1186/s13018-026-07144-0)
Supplement: Supplementary file 3 [file 13018_2026_7144_MOESM8_ESM.docx]

Definition of Fusion / Union Across Included Studies

| **#** | **Study** | **Fixation method** | **How fusion / union was defined** | **Imaging modality used** |
| --- | --- | --- | --- | --- |
| **10** | **Manchanda et al., 2020** | Intramedullary midfoot beaming | Not explicitly defined | Plain radiographs |
| **17** | **Mehlhorn et al., 2016** | Midfoot fusion bolt + lag screws | Radiographic consolidation of arthrodesis sites | Plain radiographs, SPECT/CT |
| **23** | **Pinzur & Schiff, 2018** | Circular | Fusion not used as an outcome metric. | Not assessed |
| **28** | **Regauer et al., 2023** | Screws and Plates | Fusion not assessed | Not assessed |
| **31** | **Kroin et al., 2018** | Circular | Fusion is not assessed. | Not used for fusion |
| **35** | **Richter et al., 2015** | Midfoot Fusion Bolts | Union assessed radiographically; “fused” judged by the surgeon | Plain radiographs; CT or PedCAT per local standard |
| **37** | **Eschler et al., 2015** | Internal corrective arthrodesis | Fusion not defined directly. Nonunion defined as a delay in healing evident for at least 6 months postoperatively; fusion inferred from absence of nonunion. | Plain radiographs |
| **48** | **Ford et al., 2018** | Beaming | No explicit definition. Osteotomies reported simply as "united on final radiographs" (43% united). | Plain radiographs |
| **49** | **Sammarco et al., 2009** | IM screws | Three-tier classification: complete osseous union; partial union (one joint failed to unite in an otherwise stable foot); non-union (failure to unite with recurrence of deformity). | Plain radiographs |
| **50** | **Matsumoto et al., 2015** | MAC Exfix | Union = radiographic evidence of bony consolidation at the fusion site (also used as the threshold to begin weight-bearing). | Plain radiographs |
| **51** | **Pinzur et al., 2007** | Circular | Fusion not defined and not reported as an outcome. | Not assessed |
| **59** | **Assal & Stern et al., 2009** | Medial Column Screw | Nonunion defined as a lack of radiographic signs of healing at 6 months postoperatively (fusion = presence of radiographic signs of healing). | Plain radiographs |
| **60** | **Garchar et al., 2013** | Plantar Plate | “Union” defined as a clinically stable foot with no sclerosis or gapping at the fusion site, maintenance of solid hardware, and trabeculation seen on radiographs. | Clinical stability + Plain radiograph |
| **71** | **Rios Ruh et al., 2019** | Circular | Fusion not assessed | Fusion not assessed |
| **78** | **Farber et al., 2002** | Skeletal fixator | Bony union determined by observing callus formation across the fusion site on radiographs; if callus was not clearly present, a fibrous union was assumed. | Plain radiograph |
| **82** | **Mehlhorn et al., 2020** | IFB (two types MFB, HTFB) | NR | NR |
| **84** | **Loveland et al., 2024** | IMN | “Successful fusion” based on clinical findings, CT imaging (≥50% osseous bridging), and/or radiographic consolidation (3 or 4 of 4 cortices healed). | Clinical + CT + Plain radiograph |
