# Supplementary material for: Outcomes of internal and external fixation in midfoot Charcot neuroarthropathy: a systematic review and single-arm meta-analysis
Source: J Orthop Surg Res. 2026 Jul 30;21:612. doi: 10.1186/s13018-026-07144-0 (PMC13711758; doi:10.1186/s13018-026-07144-0)
Supplement: Supplementary file 4 [file 13018_2026_7144_MOESM9_ESM.docx]

Supplementary Table: Surgical indication, timing of fusion assessment, offloading, and pedorthotic aftercare across included studies

| **Study (fixation)** | **Surgical indication & infection/ulcer status at surgery** | **Timing of radiographic fusion assessment** | **External-fixation duration / offloading (NWB)** | **Pedorthotic aftercare (type)** |
| --- | --- | --- | --- | --- |
| **Internal fixation (IF)** | | | | |
| **Manchanda et al. 2020**  *IF* | - Indication: Midfoot reconstruction for limb salvage, Sanders-Frykberg II/III - Infection/ulcer: Not separately stratified. Preoperative wound in a subset | - No protocolised timepoint - Assessed at latest follow-up (mean 67.4 weeks; range 3-17 months) | - Offloading / NWB (Non-weight bearing) protocol not reported | - NR |
| **Mehlhorn et al. 2016**  *IF* | - Indication: Non-plantigrade foot, high instability, plantar ulcer from deformity, or failed conservative care; Eichenholtz III/late II - Infection/ulcer: Preoperative ulcer 8/14; callosity 2/14; ulcer-free 4/14 | - Serial radiographs: 2 days, 6/12/16 weeks, 6/12/18/24 months | - NWB cast 12 weeks | - Orthopaedic shoes or sports shoes |
| **Regauer et al. 2023**  *IF* | - Indication: Sanders 2/3 with severe dislocation - Infection/ulcer: N/A - infected tissue / open plantar wounds were excluded | - Radiographic assessment at 6 weeks, 3/6/12 months | - CROW boot offloading for 12 weeks | - Custom orthotic shoe with pressure-optimised insoles |
| **Richter et al. 2015**  *IF* | - Indication: Reconstruction for CN/deformity with neuropathy - Infection/ulcer: Active ulcer on treated foot 5/48; remainder ulcer-free | - Radiographic assessment at 6/9/12 weeks | - Immobilisation mean 11.1 weeks (4-18); partial Weight-bearing (15 kg) ≥6 weeks; walking aids mean 12 weeks | - Foot/ankle orthosis 54%, TCC 17%, removable cast walker 15% |
| **Eschler et al. 2015**  *IF* | - Indication: Non-plantigrade alignment, high instability, manifest/impending ulcer over deformity, failed conservative care; all ≥2 Pinzur high-risk criteria - Infection/ulcer: Ulceration 38%, imminent ulceration 43%, ulcer-free 19% | - Monthly until consolidation, then yearly | - Splint then TCC; partial Weight Bearing (20 kg) 6-12 weeks; mean casting 2.7 months (1-7) | - Orthopaedic shoes |
| **Ford et al. 2018**  *IF* | - Indication: Midfoot Charcot collapse ± ulceration - Infection/ulcer: Pre-operative ulcer in a subset; predicted deep infection (all 6 deep infections in preop-ulcerated feet) | - No defined timepoint | - NR | - NR |
| **Sammarco et al. 2009**  *IF* | - Indication: Unstable midfoot deformity (progressive deformity, gross instability, bayonet deformity, recalcitrant ulcer) failing conservative care - Infection/ulcer: N/A - Ulceration or infection noted as contraindication | - Fusion judged radiographically (4-6 months) | - NWB until radiographic maturation (4-6 months) then graduated Weight Bearing in pneumatic boot | - Multidensity foam orthosis + accommodative footwear |
| **Assal et al. 2009**  *IF* | - Indication: Rocker-bottom midfoot deformity with nonhealing plantar ulcer failing CROW, or unbraceable instability - Infection/ulcer: Nonhealing ulcer 13/15; 4 with active infection treated as osteomyelitis, operated only after resolution (6-week organism specific IV antibiotics) | - Anteroposterior and lateral WB radiographs of the foot made at follow-ups, no defined f/u schedule - Mean F/U 41.5 +/- 12.3 | - Strictly NWB first 4 months then progressive Weight bearing in removable cast (~8 weeks) | - Extra-depth wide toed shoes with moulded inserts; 1 used CROW |
| **Garchar et al. 2013**  *IF* | - Indication: Lisfranc dislocation 2° to CN; chronic ulcers with bony deformity, unstable non-braceable joints, recurrent infected ulcers with bony prominence, acute displaced neuropathic fractures (adequate circulation) - Infection/ulcer: Operated in inactive phase (late stage II / III); preoperative ulcer/infection status not separately stratified | - Serial radiographs every 3 weeks postoperatively | - NWB postoperatively per radiographic/clinical appearance - Mean time to ambulation 11.68 weeks (7-20) | - Walking cast or CAM walker for 2-3 months after start of weight bearing - Longer-term pedorthotics |
| **Mehlhorn et al. 2020**  *IF* | - Indication: instable Charcot midfoot collapse — non-plantigrade alignment, high instability, impending plantar ulcer from deformity, or increasing pain; Eichenholtz II/III (acute I excluded) - Infection/ulcer: active ulceration 11/40 (25-30%); osteomyelitis not specifically addressed or excluded | - CT at 3 months - WB radiographs at 3, 4, 6, 12 months; Meary's angle at 3 & 12 months | - NWB cast 12 weeks then gradual full Weight bearing | - NR |
| **Loveland et al. 2024**  *IF* | - Indication: unstable midfoot Charcot deformity; limb salvage / avoid amputation - Infection/ulcer: Preoperative ulceration 6/30 (20%; 3 plantar medial, 3 plantar lateral); no active infection focus described | - CT at 12-16 weeks | - Postop external fixator 25/30 (or posterior splint 5/30) until fusion; removed 3-4 months - Mean time to braced Weight bearing 18.1 weeks (12-28) | - CROW boot up to 1 yr then extra-depth shoe by 1 yr (all patients) |
| External fixation (EF) | | | | |
| **Pinzur et al. 2018**  *EF* | - Indications: (1) nonplantigrade deformity + wound + osteomyelitis; (2) nonplantigrade without infection (to enable shoe wear); (3) unstable 'rocker' deformity with pain - Infection/ulcer: NR however Infection resected when present | - Radiographic fusion timepoint not reported | - EF: 3-level static circular frame - Frame duration / NWB period not specified | - Commercial therapeutic footwear + custom accommodative orthoses; CROW/AFO in poor-outcome subset |
| **Kroin et al. 2018**  *EF* | - Indication: nonplantigrade midtarsal CN deformity, no tibiotalar disruption - Infection/ulcer: Not stratified however osteomyelitis resected when present | - NR | - EF: 3-level static circular frame for 12 weeks | - Commercial therapeutic footwear; short AFO / custom shoe modification / CROW in some |
| **Matsumoto at el. 2015**  *EF* | - Indication: Progressive deformity with instability failing TCC, or rigid deformity with recurrent plantar ulcer unresponsive to orthoses - Infection/ulcer: Chronically infected ulcers 3/10 (Wagner 1-2); infections excluded | - Radiographic fusion timepoint not reported | - EF: MAC monolateral fixator removed mean 8.7 weeks (5-14) - Then NWB TCC mean 14.3 weeks; WB in TCC mean 20.9 weeks; diabetic shoe mean 25.2 weeks | - Custom diabetic shoes + insoles (10/11 feet); commercial shoes (1) |
| **Pinzur et al. 2007**  *EF* | - Indication: Eichenholtz II/III nonplantigrade midfoot deformity, high-risk (morbidly obese / immunocompromised) - Infection/ulcer: 14/26 wounds >2 cm with underlying osteomyelitis; infected bone excised intraoperatively | - Radiographic fusion timepoint not reported | - EF: neutral 3-level ring fixator; removed 8 weeks → WB TCC 4-6 weeks → pneumatic fracture boot - 30 lb WB permitted during fixation | - Commercial depth-inlay shoes + custom accommodative foot orthoses |
| **Rios Ruh et al. 2019**  *EF* | - Indication: Deformity with ulcer, osteomyelitis or instability; peripheral neuropathy; failed orthopaedic care (TCC, orthopaedic boots, insoles); all Eichenholtz III - Infection/ulcer: 8/10 cutaneous ulcers (6 lateral plantar, 2 lateral malleolus) with suspected osteomyelitis; 2/10 unstable, high ulcer risk; intraoperative osteomyelitis confirmed 7/10 (70%) | - No standardised radiographic timepoint | - EF: Static circular fixator (DOR System), 2 rings + foot base - Partial WB - 50% with crutches at 48-72 h; frame removed mean 12 weeks (8-14) - Then TCC 4 weeks → walker boot → adapted diabetic footwear | - TCC → walker boot → custom diabetic footwear once swelling resolved |
| **Farber et al. 2002**  *EF* | - Indication: Midfoot CN with collapse and ulceration, at risk of amputation; infection precluding internal fixation; failed nonoperative care (wound care, TCC, antibiotics) - Infection/ulcer: All ulcerated (Wagner 2-3 typical); osteomyelitis - 4/11 definitive on histology | - Radiograph on follow-up - Follow-up mean 24 months (12-49) | - EF: External skeletal fixator, mean 57 days (12-113; ~8 weeks); WB minimised during fixation - Then TCC mean 131 days (52-271) → WB as tolerated | - Custom moulded inserts + custom diabetic shoes ± patellar-tendon-bearing brace; no CROW used |

*NR, not reported; CN, Charcot neuroarthropathy; IF, internal fixation; EF, external fixation; IM, intramedullary; NWB, non-weight-bearing; WB, weight-bearing; TCC, total contact cast; CROW, Charcot restraint orthotic walker; AFO, ankle-foot orthosis; ATL, Achilles tendon lengthening; TAL, tendo-Achilles lengthening; CAM, controlled ankle motion; MAC, multi-axial correction; DOR, distraction osteogenesis ring; CLI, critical limb ischaemia; CT, computed tomography.*
